# A framework and workflow system for virtual screening and molecular docking

**DOI:** 10.1186/1758-2946-3-S1-P15

**Published:** 2011-04-19

**Authors:** M Schumann, M Röttig, NM Fischer, O Kohlbacher

**Affiliations:** 1 Zentrum für Bioinformatik, Eberhard Karls University of Tübingen, Germany

## 

Virtual Screening and molecular docking usually require a large number of diverse and often incompatible programs. Problems arise when different programs require dissimilar file formats, are not available due to license issues, or have to be used in very different kind of ways. Furthermore, data is commonly not stored consistently and information about which tools were applied on which datasets in which way is usually lost.

Thus, in practice, frequent changes between tools and environments are necessary as well as – usually not lossless – conversions between different formats and results obtained this way are hard to reproduce.

Here we present a framework, based on the Biochemical Algorithms Library (BALL) [1], that provides a broad range of functionality, covering all common areas of interest for virtual screening and molecular docking. It includes tools for ligand/receptor preparation, interaction scoring, docking and rescoring, as well as for performing QSAR analysis and storage of molecules in a database.

This framework thus greatly facilitates the application of virtual screening and docking and consistent storing and handling of data.

Furthermore, all tools of our framework have been integrated into the workflow management system *galaxy*[2]. This way, the programs can be used directly from a webbrowser, without any need for programming skills or installation of tools on the part of the user. Workflows can be easily generated, stored and reused, which makes successive application of tools much easier. All data sets and uses of tools are automatically tracked and logged, so that results obtained by use of this system are easy to reproduce.
